# Toxic Substances in Plastics, Micro- and Nanoplastics: Utilizing ATSDR’s Plastics-Related Toxicological Profile Tool and Mixtures Framework for Human Health Risk Assessment

**DOI:** 10.3390/toxics14050429

**Published:** 2026-05-13

**Authors:** Custodio V. Muianga, Gregory M. Zarus, Katie Stallings, Gaston Casillas, Mohammad Shoeb, Kimberly Gehle, Mohammad Moiz Mumtaz, Christopher M. Reh

**Affiliations:** 1 Office of Innovation and Analytics (OIA), Agency for Toxic Substances and Disease Registry (ATSDR), Centers for Disease Control and Prevention (CDC), Atlanta, GA 30341, USA; gmzarus@gmail.com (G.M.Z.); qan7@cdc.gov (G.C.); ywo7@cdc.gov (M.S.); 2 Our1World, 908 Carpenter St., Brunswick, GA 31520, USA; 3 Oak Ridge Institute for Science and Education (ORISE), Oak Ridge, TN 37830, USA; 4 Office of the Associate Director of Agency for Toxic Substances and Disease Registry (ATSDR), Centers for Disease Control and Prevention (CDC), Atlanta, GA 30341, USA; ebk2@cdc.gov (K.G.);

**Keywords:** toxic pollutants, microplastics, nanoplastics, chemical mixtures

## Abstract

The prevalence of plastics in the environment raises concerns about their complex and poorly understood effects on human health. Research continues to uncover more sources of exposure and wider ranges of plastics within the body. Adverse health effects have been observed in animals, but their relevance to humans remains unclear. To address the growing need for reliable toxicity assessment resources and tools to aid in the synthesis of findings and the identification of data gaps and needs, we have developed a data visualization tool to provide streamlined access to the evaluated data on the chemical impacts of plastics on human health. The Plastics-Related Toxicology Profiles Tool uses Tableau Public to organize the extracted chemical-specific information from ATSDR Toxicological Profiles, the United Nations Environmental Program’s 2023 Chemicals in Plastics Technical Report, and a literature review of relevant research in Google Scholar and PubMed. The tool organizes extracted data from 98 ATSDR Toxicological Profiles representing over 476 substances related to plastics production in 16 tabulated health outcome categories associated with plastics exposure. The chemicals are organized into four categories based on their role in plastics manufacturing. The top four health endpoints impacted by all listed substance profiles are respiratory, neurologic, hepatic, and developmental effects. More than 30% of the substance profiles affected these systems as well as other non-cancer endpoints involving the immunological, renal, and reproductive systems, as well as increased cancer risk in respiratory and hepatic systems. Most monomers negatively impact development and the respiratory system, and most metal additives affect the respiratory system. We explain how this data visualization tool combined with ATSDR’s framework for assessing health impacts from multiple chemicals could be applied to identify the target organs impacted by components of the common plastic polyvinyl chloride. Hazard quotients and index show low toxicity and health risk of components in the cured product. This data provide a valuable resource for prioritizing health risk assessments. Use of this interactive tool can enhance the ability of public health professionals to navigate the expanding literature, synthesize findings, and identify future health risk assessment and research priorities.

## 1. Introduction

The Agency for Toxic Substances and Disease Registry (ATSDR) is a federal agency mandated to assess exposures at waste sites and expand the knowledge of health effects from exposure to substances frequently found in the environment [1]. This mandate involves addressing environmental or waste-related exposures, including many plastics and their components. The agency continues to develop resources to assess exposure to chemicals associated with plastics and has recently initiated the development of tools for assessing the components within micro- and nanoplastics (MNPs) [1,2].

Plastics encompass a wide range of organic polymers, each designed for specific properties such as rigidity, strength, flexibility, or smoothness. Common polymers found in the environment include acrylic, polyethylene (PE), polypropylene (PP), polystyrene (PS), polyvinyl chloride (PVC), polyethylene terephthalate (PET), rubber, fiberglass-reinforced plastic, nylon, polymer-based paints, epoxy glues, polyurethane (PUR) foam, silicone, and polytetrafluoroethylene (PTFE or Teflon) [3]. Each polymer subcategory contains different chemicals to achieve desired properties. For instance, pigments, plasticizers, and stabilizers are often added. Metals in plastics act as stabilizers, biocides, and lubricants, while phthalates improve softness. Additives such as carbon strengthen plastics and protects them from degradation caused by sunlight [4].

Finished plastic products have been used for decades [3]. They are lightweight, sterilizable, impervious to water, pest-resistant, non-reactive with most chemicals, non-conductive, and resilient within specific temperature ranges. These properties make plastics essential for public health use, such as in toothbrushes, clothing, break-resistant beverage containers, and intravenous tubing [3,4].

People are exposed to plastics and their components from various environmental sources, including air, water, food, spices, cosmetics, and surgical equipment. MNPs primarily enter the human body via ingestion, inhalation, and dermal exposure [3,4]. Human biomonitoring has confirmed the presence of MNPs throughout the human body, including the brain, heart, liver, lungs, placenta, blood and others [5]. The production of PVC was recognized in the early 1940s to generate hazardous substances such as vinyl chloride residues, a known human carcinogen, listed in the International Agency for Research on Cancer (IARC) monographs since 1974 [6,7]. Despite their utility, there has been growing awareness of data gaps regarding the extent and potential health and environmental impacts of exposure to PVC and other plastic polymer pollution. Concerns include the waste and emissions from plastic production but also the breakdown products of finished plastics. Intentionally manufactured tiny particles designed for specific industrial and consumer applications (e.g., cosmetics and carriers for active ingredients) or the fragmentation and degradation of larger plastic items generate microplastics (MPs) [i.e., plastic particles less than 5 mm (mm) in size], and nanoplastics (NPs) [i.e., plastic particles smaller than 1 micrometer (µm) in size]. These processes are driven by mechanical, physical, and chemical stressors during the entire life cycle of plastic products, including manufacturing, daily use, and waste management [3,4,5,6,7]. MNPs can also be directly released to the environment through the abrasion of plastic materials during manufacture, usage, or repair—for example, wear and tear of rubber items such as tires, footwear, and synthetic textiles, particularly during cleaning or washing, and peeling of coatings and paint [4,5,6].

An illustrative example of plastic complexity is polyvinyl chloride (PVC) pipes used to transport drinking water. PVC starts as a viscous liquid, and chlorine cross-links the monomers to produce varying degrees of hardness, strength, and heat resistance. The coal-based manufacturing process uses mercury as a catalyst. Mercury is used in chlorine production, and approximately 3.7% is released into the environment, primarily as solid waste and byproducts like hydrochloric acid [6]. To modify PVC’s properties for the intended use, various additives are often mixed in, including:

- Plasticizers like di-n-butyl phthalate or di(2-ethylhexyl) phthalate (DEHP) for flexibility and longevity.
- Carbon black for strength and UV protection.
- Kaolin, silica (SiO_2_), and alumina (Al_2_O_3_) for strengthening.
- Titanium dioxide pigments for white coloration during compounding.
- Triarylmethane dye and coal black for ink labeling, produced from incomplete petroleum combustion, and others.

Thus, PVC is not merely polyvinyl chloride, but a complex mixture incorporating numerous substances that could pose toxicity concerns. ATSDR’s approach to assessing the toxicity of PVC and other plastic components involves evaluating them as mixtures [7,8,9].

The generation of MNPs from plastic polymer products like PVC pipes is primarily driven by physico-chemical aging processes that weaken the material, increasing its roughness, brittleness, and propensity to release particles into water. Key physico-chemical properties and environmental factors that drive this fragmentation include: increased hydrophilicity, surface degradation and roughness, carbonyl index, fragmentation of polymer molecular structure with UV radiation and thermal degradation, additive leaching, biofilms that grow in the inner pipe surfaces, and high density of PVC. Additional information can be found in Section 3.3.2. Comprehensive discussion of these factors is beyond the scope of this review. Human exposure to MNPs occurs primarily through ingestion, inhalation, and dermal contact. For all routes, the uptake mechanism depends on the size, shape, solubility, and surface characteristics, as well as biological factors such as the deposition site [7,8,9,10]. Estimates suggest daily exposure to thousands of MPs particles and fibers [3,4].

The available data suggests an accumulation of plastics of varying sizes in body tissues and organs, further complicating the assessment of health impacts from exposure. Micro- and nanoplastics (MNPs) have been detected in the lungs, blood, and lymphatics, as well as incorporated into tissues in the lungs, liver, spleen, brain, reproductive organs, and placentas [5,6,7,8,9]. Some plastics are excreted in feces, and trace levels of MPs smaller than 13 μm are eliminated in urine [10]. While some in vivo and in vitro studies have examined the effects of MNPs, their impact on human health remains unclear [6,7,8,9,10]. This research addresses the growing need for reliable toxicity assessment resources and tools to aid in the synthesis of findings and the identification of data gaps and needs. We have developed the Plastics-Related Toxicological Profiles visualization tool to organize chemical components of plastics and their respective health outcome categories. We demonstrate how to estimate the human health risks of PVC MNPs and leaching chemical components using the ATSDR framework for assessing the health impacts of multiple chemicals and other stressors to estimate human health risks.

## 2. Materials and Methods

The sections below summarize the data abstracted from ATSDR’s Toxicological Profiles (Tox Profiles) and other documents, show how this data was used to populate the Plastics-Related Toxicology Profiles tool, and explain how data was organized to create the tables reported in this manuscript.

### 2.1. Data Selection

We identified, extracted, and categorized MP data from ATSDR’s Tox Profiles and related peer-reviewed documents. Identification and selection of data sources primarily relied on the review of citations and references reported by ATSDR’s Tox Profiles and other ATSDR and NCEH-led publications on substances, wastes, and MPs [1,2,3,4,5,6,7,8,9,10].

Each Tox Profile provides a comprehensive summary of available evidence on the health effects of each substance, including human and animal studies that may translate to human contexts [1,2]. Substances are often identified for profile development, in part, by their position on ATSDR’s Substance Priority List (SPL) [11,12]. The SPL orders substances based on toxicity, occurrence, and exposure [11,12].

Tox Profiles undergo systematic review processes and are updated with new scientific evidence. Each profile addresses the title substance and associated isomers or compounds, which may be covered in one Tox Profile or grouped into separate Tox Profiles to better focus the volume of respective studies [1]. For example, the di-2-ethylhexyl phthalate profile also includes related phthalates and metabolites [1], while the lead Tox Profile reviews a broader range of lead species and compounds [1]. ATSDR also develops chemical interaction profiles by reviewing several substances commonly found in mixtures.

Tox Profiles compile information on a substance’s physico-chemical properties, uses, human exposure potential, toxicity, toxicokinetics, biomarkers, and minimal risk levels (MRLs). MRLs estimate the daily human exposure to a hazardous substance likely to pose minimal risk for non-cancer health effects. Draft Tox Profiles are reviewed by CDC/ATSDR experts, interagency reviewers, and independent peer reviewers before public commentary and finalization [1].

#### 2.1.1. Toxicological Profile Query

**Figure 1:**
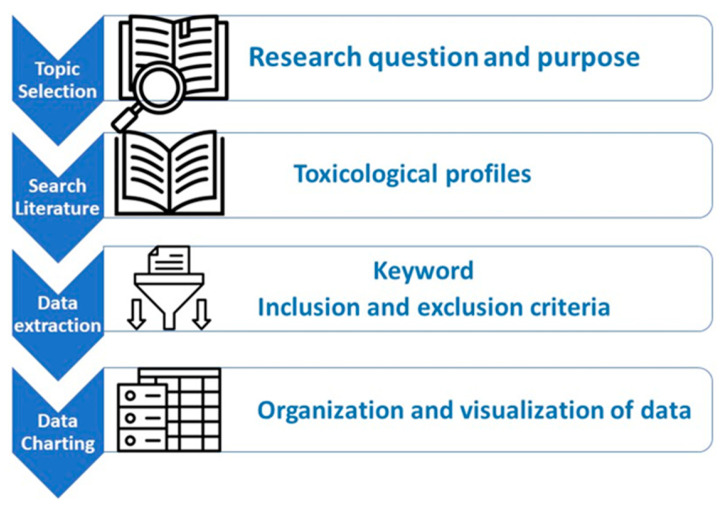
Graphical representation of Tox Profile query and charting process. Source: adapted from ATSDR [1].

A literature search for ATSDR’s Tox Profiles based on chemicals identified in the United Nations Environment Program (UNEP) and Secretariat of the Basel, Rotterdam and Stockholm Conventions (SBRSC)’s technical report on chemicals in plastics [13] was conducted from January to August 2024. At the time of this review, a total of 184 Tox Profiles had been published. Figure 1 illustrates the process used to identify candidate profiles addressing the toxicological and health effects of plastics, MNPs.

#### 2.1.2. Substance Category Abstraction

The Tox Profile query and selection processes were guided by the following inclusion and exclusion criteria:

1. Substance-specific queries were performed based on the UNEP SBRSC (2023), a technical report on chemicals in plastics [13], and were compared to all the chemicals represented in ATSDR’s Tox Profile database to identify relevant profiles.
2. Chemicals from relevant profiles were reviewed and used to group the profiles into the following categories: a. monomers, b. metals and metalloid additives, c. non-metal additives and stabilizers, and d. chemical intermediates and precursors.

#### 2.1.3. Health Outcome Category Abstraction

We reviewed Chapter 2: Health Effects of each Tox Profile and extracted information on health effects in humans and animals related to the following health outcome categories: body weight, respiratory, cardiovascular, gastrointestinal, hematological, musculoskeletal, hepatic, renal, dermal, ocular, endocrine, immunological, neurological, reproductive, and developmental effects, and increased cancer risk in various organ systems after chronic or intermediate exposure duration [1,7]. We also used the key resources of our previously published microplastic papers for MNP health effects [1,3,5,12,13]. To ensure consistency, reproducibility and reliability, there were three layers of health outcome category reviews including two extractors (KS and GC, first reviewer (CM) and the second reviewers (GMZ, MS, KG).

### 2.2. Plastics-Related Toxicological Profiles Tool

We developed a qualitative toxicological screening tool using Tableau Public ^TM^ to visualize the data extracted from ATSDR’s Tox Profiles. Tableau Public ^TM^ is a free visualization analytics platform that helps users analyze and visualize data to solve problems in an interactive way and is available to the public [2]. Tableau^TM^ facilitated the sorting of the extracted data for use in tables. The tool visually presents summaries of the plastic’s components and health outcomes extracted from the Tox Profile. Chemicals from relevant profiles were reviewed and used to group the profiles into the following categories: a. monomers, b. metals and metalloid additives, c. non-metal additives and stabilizers, and d. chemical intermediates and precursors [1].

We created tables containing the chemical components and health effects for each category. We uploaded this data into Tableau and generated interactive charts that summarized this information by category. The abstracted data focused on two major categories:

1. The plastic substance categories cited above in the inclusion/exclusion criteria (e.g., monomers).
2. The health outcome categories explained above (e.g., body weight, respiratory effects).

#### How to Use the Plastics-Related Toxicological Profiles Tool

The Plastics-Related Toxicological Profiles tool enables users to quickly identify health endpoints linked to chemicals that comprise plastic mixtures. For example, in assessing the polymer PVC, the tool allows users to evaluate potential health impacts by drawing on data from its monomer and substances involved in its polymerization process. The health endpoints identified by the tool provide a systematic starting point for investigation.

The tool is versatile and can be used for various exposure scenarios, such as:

1. Production process: identifying potential exposures to workers involved in the production of plastics.
2. Industrial releases: identifying potential uncontrolled and controlled environmental releases during the production process.
3. Product use by retailers and consumers: identifying potential exposures to chemicals in finished plastic products through usage.
4. Waste management (recycling, landfilling, and incineration) and micro- and nanoplastic (MNP) exposures: identifying potential chemical components from eroded plastic products or environmental breakdown products.

### 2.3. Assessing Health Impacts of Multiple Chemical Components of Plastic Products

We used the example of a child weighing 10 kgs (~22 lbs.) ingesting 3 µg of PVC to assess the health impacts of exposure to multiple chemical components of PVC pipe products. We used the ATSDR (2018) framework for assessing the health impacts of exposure to multiple chemicals and other stressors [14]. In this guidance document, ATSDR recommends a 3-tiered approach to evaluating exposure and health effects data to assess public health impacts in communities in the vicinity of sites of chemical or physical agent contamination. We used a component-based human health risk assessment approach to a) evaluate potential health risks posed by each individual chemical component within the PVC mixture, b) analyze the toxicity of each component and its concentration in the mixture, and then c) estimate the overall health risk associated with exposure to the PVC product. This method is particularly useful when dealing with complex mixtures like PVC, where numerous additives and plasticizers may be present, each with its own potential toxicity profile [9,13].

### 2.4. PVC Chemical Components of Health Concern as an Example

We connected data across Tox Profiles to evaluate exposures to the components of PVC pipes throughout the entire product life cycle and in environmental pollution. We selected key references for research on toxic substances in plastics, microplastics, and nanoplastics by utilizing ATSDR’s Plastics-Related Toxicological Profile tool and mixtures framework for human health risk assessment. We also defined key terms (i.e., hazardous pollutants, microplastics, nanoplastics, chemical mixtures) and key phrases using Boolean operators (AND, OR, NOT) in the PubMed and Google Scholar databases. We further screened the papers by reading titles, abstracts, results, discussions, and conclusions, prioritizing the most recent reviews with data and findings consistent with the issue in question throughout the manuscript.

## 3. Results and Discussion

This section of the paper is composed of eight subsections including the following: plastics components within Toxicological Profiles; a summary of health outcome categories reported in Plastics-Related Toxicological Profiles; estimating human health risks from exposure to plastic polymer components and residues; health hazard identification of MNPs from drinking water PVC pipes; estimating human health risks from PVC pipe chemical components; testing PVC pipes for substances associated with PVC toxicity; additional ATSDR resources to assist with communicating health risks; and limitations. In each subsection, the results are followed by a discussion.

### 3.1. Plastics’ Components Within ATSDR Toxicological Profiles

We identified 476 toxic substances associated with plastics production within 98 Tox Profiles (Table 1). Among these, 11 profiles addressed monomers, 25 addressed metals and metalloid additives, 40 addressed non-metal additives and stabilizers and 22 addressed chemical intermediates and precursors. Notably, none of the profiles directly addressed polymer plastics, but 98 profiles contained substances that are components of some polymers that were identified and are further categorized in Table 2 (a spreadsheet for chemical-specific endpoints). All Toxicological Profiles can be found at the ATSDR toxicological database, located at Toxicological Profiles|ATSDR [1].

Of the 98 Toxicological Profiles that contain components of plastic polymers, 11 Toxicological Profiles focus on at least 14 chemical monomers, which are small, single-unit molecules that are chemically bonded to form long-chain polymers, often containing reactive funcional groups such as vinyl or acrylic utilized in polymerization [15]. Some chemicals (shown in Table 1) may serve multiple purposes that span categories. However, each profile and related chemicals are assigned to only one category. For example, 4,4′-methylenedianiline can be used as an additive and stabilizer (additive and hardener) in the reaction process of forming polyurethane foams such as 4,4′-methylenedianiline diisocyanate. Other examples include phenol formaldehyde resins, commonly used in laminates, circuit boards, and construction materials. Also, m-and o-cresol mixtures can be used in the production of tricresyl phosphate and diphenyl cresyl phosphate, neutral phosphoric acid esters used as flame-retardant plasticizers for polyvinyl chloride (PVC) and other plastics, fire-resistant hydraulic fluids, additives for lubricants, and air filter oils [1,15]. Chemical subtances in ATSDR Toxicological Profiles are primarily selected from the Substaces Priority List, which identifies hazardous substances most commonly found at CERCLA NPL sites [1].

### 3.2. Summary of Health Outcome Categories Reported in Plastics-Related Toxicological Profiles [1,2,13]

Table 2 summarizes the number of substances associated with 16 health outcome categories, listed in order of frequency.

In general, health outcomes associated with exposure to plastics, MPs, NPs, and their associated chemical additives (such as BPA, phthalates, and PFAS) are multi-systemic. We stratified health outcomes into 16 categories and ranked them based on their frequency in different Toxicological Profiles (see Table 2 above). These health outcomes arise from exposure to chemicals leaching into various environmental media including water, through exposure routes of ingestion, inhalation, and dermal human contact with MNPs. From Table 2, the top five health outcome categories found in Toxicological Profiles associated with plastics exposure are hepatic, cancer (various types), renal, reproductive, immune, and gastrointestinal. Symeonides et al. (2024)’s recent umbrella review [16] reported health outcome categories from plastic- and microplastic-associated human exposure that included birth, child and adult reproductive, endocrine, child neurodeveloment, nutritional, circulatory, respiratory, skin-related, and cancers without any specific ranking. Other researchers provide ranking in terms of health effects like inflammation and oxidative stress, cardiovascular effects, digestive and gastrointestinal disorders, immune response disruption, respiratory, reproductive, developmental, cytotoxic, and genotoxic effects [16]. While there are consistencies among researchers on the targeted various health outcomes from MNP exposures, a better understanding of the cellular and molecular mechanisms of MNPs in the human body will elucidate exposure-related health effects and health risks [17].

### 3.3. Estimating Human Health Risks from Exposure to Plastic Polymer Components and Residues

We will discuss potential human exposure scenarios of PVC and how to use the Plastics-Related Toxicological Profiles tool to identify PVC-related MNPs in drinking water from PVC pipes and their associated target organs or health outcome categories.

#### 3.3.1. Potential Human Exposure Scenarios of Drinking Water, PVC Pipe Components, and Residues Case Example

MPs and NPs are increasingly found in drinking water due to the proliferation of plastics in consumer goods and infrastructures. The presence of MPs and NPs in drinking water might occur due to the prevalence and explosive growth of plastics in built environments, consumer goods, packaging, plumbing, and other sources. Depending on the environmental conditions, either MPs are released into water from the PVC pipes, which are one of the components of the drinking water distribution system (DWDS), or MPs in the water are adsorbed onto the pipes [18]. As clean water passes through aging plastic pipes, degradation mechanisms cause plastic particles to shed from pipe walls into drinking water. Surface water sources contain MPs from the breakdown of plastic waste, discharge of domestic and industrial wastewater treated in wastewater treatment plants (WWTPs), untreated domestic and industrial wastewater flowing into receiving environments, atmospheric deposition, and surface runoff. PVC and chlorinated polyvinyl chloride (CPVC) pipes may contaminate drinking water in three main ways:

- The leaching of PVC additives and residues is the main process for contaminating drinking water from the use of PVC pipes. Groundwater accounts for about 25% of drinking water worldwide—MPs can enter groundwater through leaching from heavy rains and irrigation, vertical flow via soil biological activity (e.g., burrows, adherence to organisms), seawater intrusion, and other routes, including the exchange of surface and groundwater near and downstream from rivers and streams. Also, leachates from PVC pipes disposed of in landfills can eventually contaminate soil and enter surface water and groundwater. Microplastics and nanoplastics often absorb other harmful chemical components during the seepage into groundwater. Different chemicals can be released into groundwater, which can freely enter and leave aquifers, thereby posing a chronic public health risk [15,16,17,18].
- PVC pipes can contaminate drinking water by a second process called permeation, which occurs when underground pollutants, such as gasoline leaking from a storage tank, seep through the pipe walls. In general, PVC pipes are less susceptible to permeation than other plastic pipes except at very high levels of contamination (i.e., as in a petroleum or chemical spill). Also, the oxidative properties of chlorine dioxide, a commonly used chemical in water treatment, can react with polymeric materials, potentially causing degradation and the formation of MNPs [15].
- Last, PVC and CPVC pipes may release toxic chemicals into drinking water after exposure to high heat, such as during wildfires or structure fires [above 140 °F (60 °C) for PVC and above 200 °F (93 °C) for CPVC]. Additives in plastics, including plasticizers, antioxidants, and dyes, are used to improve physico-chemical properties. Because these additives do not strongly bind to monomers, they can be easily detached, released, and discharged into the environment. MNPs leaching from aging PVC pipes used in drinking water distribution systems pose potential health risks, including the possibility of physico-chemical particle toxicity and chemical toxicity [15]. Released contaminants from thermally damaged PVC pipes include carcinogens such as the mixture of benzene, toluene, ethylbenzene, and xylenes (BTEXs), known to be associated with increased overall cancer risk and chemicals that cause long-term water quality issues [19].

The chemical composition of PVC pipes used in drinking water varies widely depending on the standards and certification process of each country and manufacturer, as well as the environmental conditions where the pipes are used. Some chemical substances associated with PVC include vinyl chloride, di-n-butyl phthalate, diethyl phthalate, DEHP, aluminum, silicates, titanium, triarylmethane, cobalt, mercury, cadmium, lead, tin, and carbon black [15,19,20].

#### 3.3.2. Physico-Chemical Properties and Processes Relevant for Generating MNPs from PVC Pipes

The generation of MPs and NPs from PVC pipes is driven by a combination of inherent physico-chemical properties such as high brittleness, low chemical reactivity, and susceptibility to dehydrochlorination and external stressors, including hydraulic shear, UV exposure, and disinfectant agents. The aging process, particularly in drinking water distribution systems, causes the inner pipe surface to crack, peel, and release plastic particles, with stagnant water conditions significantly accelerating this release [16,17,18,19].

MNPs can leach from PVC pipes into drinking water systems, influenced by factors like pH, chlorine levels, pipe aging, and continuous use temperatures. Prolonged stagnation of water in pipes can lead to higher concentrations of leached particles [20]. Also, microorganisms in biofilms can contribute to the degradation of PVC pipes, which releases MNPs and creates surfaces where pathogens can attach (e.g., legionella and certain mycobacterium species). These biofilms, the plastic particles, and the attached pathogens can then contaminate drinking water supplies, posing a risk to consumers, as well as vectors carrying pathogens to consumers. Advanced technologies applied at the design and manufacturing phases of PVC production are the most effective strategies to prevent biofilm build-up in drinking water systems. For those PVC pipes already in drinking water distribution systems, preventing and controlling biofilm build-up can be achieved by using a combination of strategies, including maintaining high water flow to prevent stagnation, adequate water disinfection, regular flushing of pipes, and removing sediments and nutrients inside pipes [18,19].

Aging, out of range service life, and exposure of PVC pipes to temperatures higher than the maximum continuous service temperature [standard PVC is 140 °F (60 °C) and CPVC is 210 °F (99 °C)] can cause leaching. Leaching from disposal facilities and from other piping components is a documented concern, particularly involving contaminants from both the pipe material itself and the solvents used to join them [15,16,17]. For example, larger-diameter PVC pipes are commonly joined using a gasket seal made of styrene butadiene rubber, from which additional VOCs, phenolic compounds, styrene, and methylene chloride may leach into water. Smaller-diameter PVC and CPVC pipe joints and segments are commonly joined using solvent-based cements and primers such as Weldon 724 CPVC or Weldon 710 PVC, respectively (composed of polyvinyl chloride resin, cyclohexanone, acetone, methyl ethyl ketone, and tetrahydro furan), where, after solvent evaporation, some residuals may leach into water [15,17].

#### 3.3.3. The Aspect of Shape and Size of Microplastics

The type of polymer and its manufacturing process have a major influence on the size and shape of resulting MNPs. The shape and size of MNPs play a crucial role in their toxicity and in the estimation of human health risks from exposure, as they dictate the capacity of tissue penetration, translocation, and toxicological interaction [18]. Nanoscale particles can more easily enter cells and organs, and their specific shape influences how they interact with biological systems. To date, multiple studies suggest that, for MNPs, the number of particles, particularly those below a certain size (i.e., particles less than 2.5 µm), is more significant for potential health effects than the total particle mass [3,4,5,6,7,8,10,13,15,16,17,18].

While most MNP studies do not fully address it, size has a major impact on associated health effects. Studies of workers, animals, and patients with plastic implants have provided evidence to suggest unique toxicity due to the forms, shapes, and sizes of MNPs [18]. Lung effects are associated with size, form, and type; liver effects were associated with type; and immune effects appear to be more affected by size and shape. Studies of liver, lung, and kidney cells show a similar proliferation effect based on size, yet drastic metabolic differences [19]. Exposing polystyrene (PS) microplastics (1 µm) to human embryonic kidney (HEK 293) and human hepatocellular (Hep G2) cell lines was shown to cause a major reduction in cellular proliferation but no significant decrease in cell viability [19]. In another study by Goodman et al. (2022) [21], human alveolar A549 cells were exposed to PS MPs of 1 µm and 10 µm; both sizes caused a significant reduction in cellular proliferation but again showed little cytotoxicity. PS MP particles of 1 µm and 10 µm were shown in experimental exposure scenarios to adversely affect embryonic brain-like tissue development in forebrain cerebral spheroids [21].

The literature examining potential cellular impacts of MP exposure is growing rapidly. This is highlighted by the fact that Khan and Jia (2023) were able to produce a 43-page review of these impacts showing, among other things, cellular organelle localization of MNPs of varying sizes, the potential underlying uptake mechanisms, and possible toxicity pathways involving reactive oxygen species [22].

Implanted plastic, ceramic, and other materials indicated that the shapes and sizes of the capsules affected absorption, more so than the material. Implanted capsules as large as 2 mm produced a tissue response [3]. Immune response is more affected by the larger microplastics than the smaller nanoplastics [23,24], whereas the lungs appear to be more affected by smaller particles [25]. Yet, some particle types produce lung effects despite the size differences of 1 micron vs. 10 microns [19,25]. Further research on the magnitude and significance of size and shape of MNPs in the development of health effects will enhance knowledge of MNP toxicity.

Adsorption/Absorption—Both the adsorption of external contaminants and the absorption (leaching) of internal additives are major concerns with PVC plastic pipes. Numerous studies have shown that PVC can act as a carrier for toxicants like heavy metals and organic pollutants, and that additives such as phthalates and metals stabilizers can migrate to the plastic and into the water and the human body [8,13,15,16,19,20]. Our PVC example touches on the many potential health risks associated with metals’ adsorption but also illustrates the smaller relative health risk when compared to exposure to PVC-related phthalates. Further research is needed for a better understanding of both physical and chemical migration processes of MNPs with a particular focus on developing advanced PVC materials that prevent the migration of internal additives and inhibit the adsorption of external environmental pollutants.

### 3.4. Health Hazard Identification of MNPs from Drinking Water PVC Pipes

Using the Plastics-Related Toxicological Profiles tool, researchers can generate a table summarizing these substances and the health endpoints associated with them. Table 3 is an illustrative example showing a select number of associated health outcome categories associated with PVC-related substances. An exhaustive list of the chemical components is outside of the scope of this paper [20].

From Table 3, one could pursue (for example) a qualitative or semi-quantitative assessment for workers exposed to chemicals, or communities exposed to components of PVC or CPVC MNPs. The tool can be used to rank the substances according to toxicity, sum the risks, and identify the uncertainties which remain in understanding total risks associated with exposure to plastics mixtures [14,15,20].

### 3.5. Estimating Human Health Risks from PVC Pipe Chemical Components

Studies have reported the current maximum MPs in tap water as 930 particles per liter (Particles/L) [17,18]. Seventeen polymer types were identified for MPs in bottled and tap water, where PVC accounted for 17%, cellulose 68%, and other polymers for less than 5% individually. Using such reports, it is possible to estimate the mass of ingested microplastics as well as estimate the daily intake of microplastics from drinking water [18,22].

Depending on the concentrations of contaminants in air, water, or food, one can derive doses and compare them to the minimal risk levels (MRLs) established by ATSDR. MRLs help public health professionals identify contaminants and potential health effects of concern at hazardous waste sites. Acute-duration MRLs apply to exposures lasting 1–14 days, intermediate-duration MRLs to exposures of 14–364 days, and chronic-duration MRLs to exposures exceeding 365 days.

For example, concerns regarding PVC MNPs in food or drinking water could be evaluated using the MRLs detailed in Table 4 [1,11,14] and the hazard quotients in Table 5.

Table 4 can be used to prioritize the level of concern for PVC components based on their toxicity. For example, vinyl chloride, DEHP, and cadmium have low MRLs (i.e., considerably protective to humans) and offer good starting points for evaluation. By using relative fractions and relative toxicities, we can demonstrate that DEHP will often drive the chemical toxicity of PVC MNPs. Additionally, some adsorbents such as aluminum, which is found naturally in the environment and adheres to MNPs, could be assessed using ratio techniques. Of the three common phthalates, DEHP has the lowest MRL value and could be the most toxic component in PVC. Henkel et al. (2019) reported that phthalates like DEHP typically make up 35% of the PVC by weight or more [26].

**Hypothetical Exposure Scenario for Worked out Example**

Chia et al. (2025) [27] characterized MP exposure in children, from infants to adolescents. Major sources include milk and infant formulas for infants, plastic toys mostly for toddlers, and adolescents exposed to MPs through consumption of contaminated food with plastics and use of food packaging. All children age groups were also exposed to MPs through water and air sources. Children are highly vulnerable to phthalate exposure from PVC materials, which leach chemicals like DEHP into drinking water, household dust, and, for infants, through mouthing toys [27]. We could estimate the daily ingested mass (i.e., the total quantity consumed) of PVC MNPs, bonded or non-bonded additives or residues leached in drinking for all ages. Oral dose (oral exposure dose) is calculated by normalizing the ingested mass by body weight (BW) and exposure factor (exposure frequency) (EF), allowing for estimating health risks across different populations (e.g., children vs. adults).

$$Dose=\frac{\left(Ingested\;PVC\;mass\times \%\;Phthalate\right)\times Ingestion\;Rate\times Exposure\;Factor\;}{Body\;Weight}$$

We estimate how much PVC a 10 kg child (i.e., approximately one year old) could be exposed to at levels exceeding the intermediate oral MRL duration dose of 0.1 µg/kg/day below, assuming the child weighs 10 kg:

$$Dose=\frac{(Ingested\;PVC\;mass\times \%\;Phthalate)\;}{Body\;Weight}=\;\frac{3\;\mu g\;\times 35\%}{10\;\mathrm{kg}}=0.1\;\mu g/kg$$

Hazard Quotient (HQ) = Dose/MRL = 0.1 µg/kg/0.1 µg/kg = 1

Thus, 1 µg of PVC could contain 0.35 μg of DEHP. Therefore, for example, a child weighing 10 kg could exceed the intermediate-duration MRL dose of 0.1 µg/kg/day by ingesting approximately 3 ug of PVC assuming the same time period of intermediate-duration exposure (15–365 days), which means continuous exposure of seven days per week and 365 days per year, resulting in an EF of 1. No chronic-duration oral MRL was derived for DEHP due to insufficient data [1].

Applying these equations for the possible percentages of other PVC components shows that other components contribute much less to the toxicity of PVC (Table 5). One can sum the HQs into a single hazard index (HI), which in this case is not much more than the HQ for DEHP. Table 5 summarizes the above dose and hazard quotient assessments assuming a 3 µg mass of PVC leached from old or damaged PVC pipes used in an older water distribution system network in a small town. An explanation of the assumptions follows.

In this example the HI is made up almost entirely of the HQ for DEHP. However, PVC could be made of other, less toxic phthalates and at lower concentrations, which could increase the relative rankings of the other components. While humans are exposed to hazardous chemicals associated with PVC pipes and their residues through drinking contaminated water or water used for cooking and beverages [8], quantitative exposure estimates are still scarce. Additional studies are greatly needed to obtain an accurate estimate of exposure.

PVC production results in the creation of several highly toxic chemicals including dioxin, ethylene dichloride, and vinyl chloride. PVC products leach phthalates and other chemicals into the environment that are, in turn, ingested by consumers and have been associated with diseases such as asthma and increases in developmental problems in children. At the end of the product’s life, incineration of PVC creates dioxin and other toxic byproducts [8,15].

Drinking water is one of the main ways microplastics enter the human body. When considering the potential hazards posed by microplastics in drinking water, it is important to take into account the following factors: firstly, the physical hazards posed by the particles; secondly, the complexity of their chemical composition, which includes but is not limited to monomeric components, additives, and various contaminants adsorbed on the surface of the particles; and finally, the possibility of biofilm on microplastics. Other relevant physico-chemical properties include particle size, surface area, morphology, particle charge, hydrophobicity, and interactions with environmental additives (adsorption and absorption). These properties dictate their behavior in the environment and may influence the toxicity to living organisms. Particle properties and biological factors may affect the cellular uptake of a particle, and consequently its capacity to exert adverse health effects [19,20,21,22].

Vinyl chloride: In cured, finished PVC, the amount of residual, unreacted vinyl chloride monomer is usually low, as most of it is heated off to achieve less than 2 ppb for water use. Leaching tests have identified less than 0.3 ppb vinyl chloride from PVC used for water. Assuming the 3 ug MNP mass consumed by the approximately one-year child with 10 kg (as detailed above), we find that the dose of uncured vinyl chloride will be several hundred times less than the oral chronic-duration MRL for vinyl chloride (3 µg/kg/day) [1,19,20].

Cadmium: Cadmium has very low MRLs. PVC studies suggest that cadmium could make up 0.001% of the PVC by weight. Thus, following the exposure scenario of an approximately one-year-old child with 10 kg, 3 µg of PVC could contain 0.00003 μg of cadmium, which represents a dose several thousand times less than the oral chronic MRL for cadmium (0.1 µg/kg/day) [1,20,28].

Aluminum: While aluminum is much less toxic than cadmium, it is abundant in the environment. It is sometimes used to wrap PVC pipes, and aluminum fragments can adhere to PVC MNPs during environmental degradation. The assessment of exposure measurements could follow the examples of DEHP and cadmium. Alternatively, a ratio technique could be used to determine the influence of aluminum on the overall toxicity. The oral chronic MRL for aluminum of 1 mg/kg/day (1000 µg/kg/day) is 10,000 times higher than that for cadmium (0.1 µg/kg/day) and 10,000 times higher than DEHP (0.1 µg/kg/day). Thus, a PVC MNP would need to comprise 20% of aluminum to impart toxicity as significant as that of cadmium. Furthermore, if PVC comprised 60% aluminum, it would be more than 55,000 times less than the relative hazards associated with DEHP [1,8,15,20,28].

Other components of PVC and other MNPs: The other PVC constituents appear to be less toxic, except for lead. While there is no identifiable safe blood lead level, it is often hard to discern the contribution of lead from plastic (like most PVC) in the environment due to other lead sources. Lead in some paints (which are plastics) could be as high as 10%, which is much higher than what is found in most other plastics [29]. Many countries continue to use lead paint, and flakes from lead paint chips continue to enter the environment. These include lead–PVC paints that are sometimes used to color imported toys [15,20]. ATSDR has investigated several communities that have been exposed to lead. In one community with older homes containing lead paint, they found that 56.7% (CI:56–57.4%) of the children had blood lead levels higher than 5 ug/dL [30]. Lead is very easily measured and could be analyzed independently of the need to characterize other constituents in MNPs [31].

The estimated HQ for specific selected components and overall HI less than 1 was consistent with the human health risk assessment performed by other researchers [16,17,31] on the ingestion of groundwater containing PVC MPs and the plasticizer DEHP. Further research is needed to incorporate additional components using more stringent and sophisticated tools such as the application of probabilistic risk assessment [32].

The EPA’s Integrated Risk Information System (IRIS) database is a premier federal source containing in-depth, peer-reviewed, toxicological assessments for over 540 chemical substances, focusing on hazard identification and dose–response, including oral reference doses and inhalation unit risks. The IRIS toxicity values support EPA regulatory decisions for environmental contaminants and are essential for risk assessment [33]. Because the IRIS database is approximately twice as large as the ATSDR Toxicological Profile database, it is likely that the IRIS database has assessed more monomers [1,33].

Future work is needed to document monomers, olygomers, polymers, their additives, and other polymer-associated chemicals with toxicity data available to continually build the Plastics-Related Tox Profiles tool in support of a stronger or more comprehensive human health risk assessment framework for MNPs. Recently, Nyambego et al. (2025) [34] proposed a data synthesis framework of additives and polymer-associated chemistries (APACs), an approach for risk-based screening and prioritization of APACs that focuses resource intensity on higher-risk scenarios. As a case study, they analyzed more than thirteen thousand APAC chemicals found in the UNEP Chemicals in Plastic Report and found that 66.9% of those chemicals had sufficent publicly available data to perform downstream human health risk assessment [13,34].

### 3.6. Testing PVC Pipes for Substances Associated with PVC Toxicity

Under the NSF/ANSI/CAN 61 Testing and Certification standard for drinking water system components, NSF tests PVC pipes for the following contaminants: volatile organic compounds (VOCs), phenolics, residual vinyl chloride monomer (RVCM), regulated metals including tin, and any other potential contaminants identified during the formulation review [15,16]. Based on NSF Testing and Certification standards, PVC pipes and fittings certified by NSF do not contain lead and this is verified via lead content testing per NSF/ANSI/CAN 372. RVCM is tested at least twice a year. Also, rigid PVC pipes and fittings certified by NSF do not contain phthalates or phthalate plasticizers. All NSF-certified PVC pipes are tested for metals at least once per year. The total amount of extracted tin (in organotin) is evaluated against pass/fail criteria based on the type of tin stabilizer in the PVC product (e.g., mono/dibutyl tin, mono/dimethyl tin, mono/dioctyl tin, etc.) [15]. These standards, certifications and practices are likely met in the United States where there is a well-established robust standards and certification program for drinking water treatment chemicals (NSF/ANSI 60). The NSF/ANSI 60 standard also establishes minimum health effect requirements for chemicals and materials that come into contact with drinking water, ensuring public health, safety, and purity [15]. The use of lead and phthalate additives in PVC products manufacturing is determined by country-specific regulations and standards. Stricter standards and outright bans have been established in North America and in the European Union, especially in products for children. However, studies and reports highlight the continuing prevalence of lead and phthalates in PVC products, including PVC pipes used for drinking water distribution in many regions around the globe. Additional testing of PVC pipe chemical components, exposure, and toxicity assessments using more sensitive and sophisticated analytical methods are needed to design robust animal laboratory toxicological and epidemiological studies for human health risk assessment [8,13,15,16,17,20,22,32].

### 3.7. Additional ATSDR Resources to Assist with Communicating Health Risks

There are ToxFAQs™ and ToxGuides™ for many plastics-related substances with Tox Profiles including di-n-butyl phthalate, DEHP, vinyl chloride, cadmium, aluminum, cobalt, lead, tin, mercury, and chlorine discussed in this paper. The ATSDR ToxFAQs™ series provides user-friendly information from the ATSDR Tox Profiles. Each fact sheet serves as a quick and easy-to-understand resource. Answers are provided to the most frequently asked questions (FAQs) about exposure to hazardous substances found around hazardous waste sites and the effects of exposure on human health [1,14]. The ATSDR ToxGuides™ are quick reference guides on the chemical and physical properties, sources of exposure, routes of exposure, MRLs, children’s health, and health effects. The ToxGuides™ also discuss how the substance might interact in the environment. They were developed by summarizing the key information from the corresponding Tox Profile [14].

The ToxFAQs™ and ToxGuides™ are available in both HTML and PDF format for each published Tox Profile, which provides the familiar two-page print version widely used at community meetings [1,14].

### 3.8. Limitations

As discussed above, a major limitation in assessing micro- and nanoplastic exposures is that no polymers have been profiled by ATSDR. As previously reported in the Toxicological Profiles and elsewhere, monomers and other substances in the polymer mixture can be used to identify potential chemical toxicity and genotoxicity, but other properties of the polymer become important when items break down into MNPs. Some of these MNPs pass through the body unchanged. However, MNPs can be inhaled or ingested and are small enough to translocate to various target organs, including the brain, where they pose potential health risks. Their ability to cross the blood–brain barrier (BBB) is an area in significant need of research, particularly regarding how they act as carriers for other environmental toxins [16,20,22].

The lack of ATSDR’s polymer-specific profiling is partially due to the lack of many polymers being characterized on hazardous waste sites. The SPL prioritization process affects topic selection for the Tox Profiles. Polymers are seldom sampled, due to the lack of approved sampling methodologies and the limited use of the newer emerging technologies on hazardous waste sites [35]. Should approved methodologies be applied at waste sites and specific polymers detected, these polymers can be ranked on the SPL and selected based on priority. Nevertheless, many of these cured polymers are much more stable than the monomers or components, and reactivity is a major determinant of toxicity [19,20].

Currently, we only found two polymers among 275 listed in the ATSDR 2022 Substance Priority List, including styrene–acrylonitrile polymer (ranked 456) and acrylic acid polymer (ranked 716). Their relatively low ranking is due to their limited frequency of being detected at hazardous waste sites and in part due to their low toxicity [35]. Of these two, only styrene–acrylonitrile appears to be sufficiently toxic to warrant possible concern should it be increasingly found [36,37]; yet, some of the information could be found separately in the acrylonitrile and styrene Tox Profiles [1,36,37]. ATSDR continues to develop new profiles and update older ones. As a result, the Plastics-Related Tox Profiles|Tableau Public tool will continue to include more substances and updated information on potential health effects [1,2].

## 4. Conclusions

We present a novel tool that efficiently identifies ATSDR’s toxicological resources relevant to plastics-related components. The Plastics-Related Tox Profiles tool categorizes these components, streamlining the identification of potential health outcomes and prioritizing substances based on their toxicity. By applying this tool with ATSDR’s framework for assessing human health risk from exposure to multiple chemicals, we analyzed the relative toxicity of PVC components using their corresponding Tox Profile data, identifying DEHP as the dominant risk component. This example also demonstrates the importance of actual field measurements of plastic constituents at the point of exposure as well as the utilization of chemical-specific MRLs for estimating human health risks from exposure to various PVC pipe mixture components using ATSDR’s framework for assessing the health impacts of exposure to multiple chemicals and other stressors.

We present numerous ways this new data visualization tool could be used in streamlining access to rigorously evaluated data on the health impacts from exposure to microplastics. The Plastics-Related Toxicological Profile tool organizes health endpoint data from ATSDR’s Toxicological Profiles with the integration of ATSDR Substance Priority List rankings and polymer use information to help contextualize chemical hazards within plastics production.

Application of the tool in focused screening and data organization of health outcomes could be made to specific populations such as workers; it could be used to prioritize the risk assessment of components found in the environment, and to refine sampling and analytical methodologies. Given the destructive nature of analytical methods and challenges in isolating microplastics, the tool’s risk-based hierarchy allows investigators to prioritize their analyses effectively. Challenges are associated with exposure metrics and heterogeneities of MNP characteristics including diverse shapes, sizes, polymer types, various chemical additives, colors, and particle charge, which complicate the risk assessment.

Key Conclusions:

- Plastics Industry: The chemical toxicity of many plastic components is well-understood. The tables and tools provided in this manuscript support evaluations of workers and community exposures related to production and environmental waste releases from industrial activities.
- Weathering Plastics: While toxicity studies of cured polymers are less abundant than those of their components, leaching studies show that the chemicals released during polymer weathering are generally less toxic than the original components. Phthalates dominate the chemical toxicity of many MNPs like PVC, while lead, which has no identifiable blood lead level considered safe, drives the toxicity of leaded paint MNPs.
- Physical Characteristics: A significant data gap remains regarding the role of size and shape in MNP toxicity. The breakdown of polymers and plastics may still present health risks due to the physical properties of the particles, independent of their chemical composition.
- Human Health Risks: Current studies predominantly focus on commercially available primary MPNs such as PS, PE, PVC, acrylic, and aramid. The tables and tools described in this manuscript provide a foundation for prioritizing toxicity research on MNPs, and as an example, we demonstrate how to assess and prioritize risks associated with PVC MNPs. The tool and estimations of HQs and HI of key chemical components leached from PVC pipes are critical for developing human health risk assessment frameworks for MNPs as emerging environmental hazards.
- Mitigation strategies: Mitigation strategies—including policies for sustainably using plastics and reducing environmental pollution, standardized sampling and analytical methods, and robust long-term animal laboratory toxicological and human epidemiological studies—are warranted.

## 5. Future Research Considerations

The tabular resources and associated tool in this manuscript enable researchers to prioritize ATSDR Toxicological Profile data on plastics and exposure-associated health outcome categories for evaluating substances in plastic-specific mixtures.

As MNPs become better characterized in air, water, and food, these tables can be sorted to support a more comprehensive mixture assessment that addresses specific exposure routes.

Field sampling designs can leverage the Plastics-Related Tox Profiles tool to optimize analytical techniques, while toxicological investigations can focus on identifying and analyzing exposure-associated health outcomes as part of human health risk assessment and to enhance the development of preventive public health strategies to minimize toxicity and human health impacts from MNP exposure.

The consolidation of microplastics research through the use of an interactive, publicly accessible tool can enhance the ability of public health professionals to navigate the expanding literature, synthesize findings, and identify health risk assessment and future research priorities.

## Figures and Tables

**Table 1:** ATSDR Toxicological Profiles known to contain data on chemical components * of plastic polymers [1,3].

Monomers	Metal and Metalloid Additives	Non-Metal Additives and Stabilizers	Chemical Intermediates and Precursors
11	25	40	22
Acrylamide (1) Acrylonitrile (1) 1,3-Butadiene (1) 1,1-Dichloroethene (1) Ethylene Glycol (1) Hexamethylene Diisocyanate (HDI)—(1) 4,4′-Methylenedianiline (1) Styrene (1) Toluene Diisocyanate and Methylene Diisocyanate (5) Vinyl Acetate (1) Vinyl Chloride (1)	Aluminum and Compounds (12) Antimony and Compounds (5) Arsenic (10) Asbestos (5) Barium (8) Beryllium (2) Boron (8) Cadmium (10) Chromium (19) Cobalt (11) Copper (3) Lead (12) Manganese (7) Mercury (6) Molybdenum (6) Nickel (4) Selenium (2) Silica (11) Silver (3) Strontium (5) Tin and Compounds (19) Titanium Tetrachloride (1) Tungsten (3) Vanadium (2) Zinc (8)	Aldrin and Dieldrin (2) Ammonia (2) Atrazine (1) Bis(chloromethyl) ether (BCME)—(1) Chlorinated Dibenzo-p-dioxins (2) Chloroethane (1) Chloroform (1) Chlorophenol (5) Creosote (2) Cyanide (2) 1,2-Dichloroethene (3) 1,2-Dichloropropane (1) 1,2-Diphenylhydrazine (1) 3,3′-Dichlorobenzidine (2) 2,4-Dichlorophenoxyacetic Acid (2,4-D)—(1) DDT, DDE, and DDD (6) DEHP—(1) Di-n-butyl Phthalate (1) Di-n-octylphthalate (DNOP)—(1) Dinitrocresols (1) Endrin (3) 2-Hexanone (1) Hexachlorobutadiene (1) Hexachlorocyclohexane (HCH)—(4) n-Hexane (1) Isophorone (1) Methyl-tert-butyl Ether (MTBE)—(1) Mirex and Chlordecone (1) n-Nitrosodi-n-propylamine (1) N-Nitrosodimethylamine (NDMA)—(1) N-Nitrosodiphenylamine (1) Naphthalene, 1-Methylnaphthalene, and 2-Methylnaphthalene (3) Nitrobenzene (1) Pentachlorophenol (2) Perfluoroalkyls (PFAS)—(9) Polybrominated Biphenyls (PBBs)—(3) Polybrominated Diphenyl Ethers (PBDEs)—(13) Polychlorinated Biphenyls (PCBs)—(142) Polycyclic Aromatic Hydrocarbons (PAH)—(22) 1,1,1-Trichloroethane (1)	Acetone (1) Acrolein (1) 2-Butanone (1) Benzene (1) Bis(2-chloroethyl) ether (BCEE)—(1) Carbon Disulfide (1) Chlorine (1) Chlorobenzene (1) Chloromethane (1) Cresols (4) ** 1,2-Dibromoethane (1) 1,2-Dichloroethane (1) Ethylbenzene (1) Ethylene Oxide (1) Formaldehyde (1) 4,4′-Methylenebis(2-chloroaniline) (MBOCA)—(1) Methylene Chloride (1) Phenol (1) 1,1,2-Trichloroethane (1)1,2,3-Trichloropropane (1) Toluene Xylenes

* Many of these chemical components can serve multiple purposes that span categories. ** Cresols are primarily used as chemical intermediates to synthesize phenolic resins which are used in the production of various polymers with desired properties like heat resistance and strength. Plastic examples include phenol formaldehyde resins, commonly used in laminates, circuit boards, and construction materials.

**Table 2:** Summary of Toxicological Profile data by health outcome category associated with plastics exposure.

Hierarchy of Health Outcome Categories Containing Data Related to Plastics Exposure	Number of Tox Profiles Containing Data per Health Outcome Category
Respiratory	56
Neurological	53
Developmental	53
Hepatic	53
Cancer	39
Renal	38
Reproductive	35
Immune	32
Gastrointestinal	26
Hematological	23
Dermal	18
Endocrine	17
Body Weight	17
Cardiovascular	15
Ocular	14
Musculoskeletal	6

**Table 3:** PVC-related substances and select associated health outcome categories.

PVC Component	Repro.	Resp.	Develop.	Neuro	Renal	Hepatic	Immuno.	Gastro.	Hemato	Dermal
Vinyl chloride										
Chlorine										
Di-n-butyl phthalate										
Diethyl phthalate										
Aluminum										
Cadmium										
Cobalt										
Lead										
Mercury										
Silicates										
Tin										
Titanium										
Carbon Black *										
Triarylmethane *										

Repro. = reproductive effects; Resp. = respiratory effects; Neuro = neurological effects; Develop. = developmental effects; Hepatic = hepatic effects; Renal = renal effects; Gastro. = gastrointestinal effects; Immuno. = immunological effects; Dermal = dermal effects; Hemato = hematological effects. * There are no profiles for triarylmethane and carbon black. Dark blue indicates that the form of the substance is associated with the health effect. Light gray means that health effects associated with titanium tetra chloride might apply. **Source:** Adapted from the ATSDR (2025) [1,2,8,11,12,14].

**Table 4:** ATSDR MRLs of constituents found in the environment associated with PVC pipes.

Oral Route of Exposure MRL (mg/kg/Day) ^†^			
Selected PVC Product Components	Acute Duration (mg/kg/Day) ^†^	Intermediate Duration	Chronic Duration
Di-n-butyl phthalate *	0.5	-	-
DEHP *	0.003	0.0001	-
Diethyl phthalate *	7	6	-
Vinyl chloride	-	-	0.003
Mercury	-	-	0.0001 ^#^
Cadmium	-	0.0005	0.0001
Tin	-	0.3	-
Lead **	-	-	-
Aluminum	-	1	1
Cobalt	0.03	0.03	-

* Rigid PVC does not contain phthalates; semi-rigid: contains about 10–26 parts per hundred resins of plasticizers (i.e., typically phthalates). ** The CDC has determined that there is no safe level of lead exposure in children. Because the lowest blood lead levels are associated with serious adverse effects (e.g., declining cognitive function in children), MRLs for Pb have not been derived. **^†^** mg/kg/day = milligrams of substance per kilograms of body weight per day ^#^ Methyl mercury (-) = none indicates the lack of an MRL. **Source:** adapted from ATSDR [1,2,6,14].

**Table 5:** Dose and hazard quotients of polyvinyl chloride (PVC) components *.

Substance	Percentage in PVC (%)	Child’s Body Weight (kg)	Calculated Dose (µg/kg)	MRL (µg/kg/Day)	Hazard Quotient
DEHP	35	10	0.1	0.1	1.0
Vinyl chloride (15%)	15	10	0.0045	3.0	1.5 × 10^−3^
Vinyl chloride (0.3 ppb)	3 × 10^−8^	10	9 × 10^−9^	3.0	3 × 10^−9^
Cadmium	0.001	10	3 × 10^−6^	0.1	3 × 10^−7^
Aluminum (20%)	20	10	0.06	1000	0.00006
Aluminum (60%)	60	10	0.18	1000	0.00018
Hazard Index	The hazard index (HI) is the sum of the hazard quotients (HQ)				>1

* We excluded lead. Lead cannot be evaluated in this manner as there is no safe identifiable blood lead level. The CDC has determined that there is no safe level of lead exposure in children. Because the lowest blood lead levels are associated with serious adverse effects (e.g., declining cognitive function in children), MRLs for Pb have not been derived.

## Data Availability

No new data were created or analyzed in this study. Data sharing is not applicable to this article.
